# Metacognitions Are Associated with Subjective Memory Problems in Individuals on Sick Leave due to Chronic Fatigue

**DOI:** 10.3389/fpsyg.2016.00729

**Published:** 2016-05-13

**Authors:** Henrik B. Jacobsen, Julie K. Aasvik, Petter C. Borchgrevink, Nils I. Landrø, Tore C. Stiles

**Affiliations:** ^1^Hysnes Rehabilitation Center, St. Olav's University HospitalTrondheim, Norway; ^2^National Competence Center for Pain and Complex Disorders, St. Olav's University HospitalTrondheim, Norway; ^3^Department of Circulation and Medical Imaging, Norwegian University of Science and TechnologyTrondheim, Norway; ^4^Clinical Neuroscience Research Group, Department of Psychology, University of OsloOslo, Norway; ^5^Department of Psychology, Norwegian University of Science and TechnologyTrondheim, Norway

**Keywords:** metacognition, chronic fatigue, memory, attention

## Abstract

**Background:** Subjective cognitive impairments are frequent, but poorly understood in patients with chronic fatigue. We hypothesized that maladaptive metacognitive beliefs at baseline were associated with baseline subjective cognitive impairments, that they predict subjective cognitive impairments at treatment termination, and that a reduction in maladaptive metacognitive beliefs was associated with less subjective cognitive impairments at treatment termination, independent of changes in fatigue, pain, insomnia, depression, and anxiety.

**Methods:** In this non-controlled study, patients (*n* = 137) on sick leave due to chronic fatigue received a 3.5-week inpatient RTW rehabilitation program. Of these patients 69 (50.4%) was referred with a ICPC-2 diagnosis of chronic fatigue. Patients completed questionnaires about metacognitive beliefs, somatic complaints, psychological complaints, and cognitive impairments before and after treatment. To test the hypotheses we performed paired *t*-tests of change, as well as seven hierarchical linear regressions.

**Results:** Results showed that baseline maladaptive metacognitive beliefs were significantly associated with subjective cognitive impairments at baseline, controlling for symptoms. Score on baseline metacognitive beliefs did not predict impairments post-treatment. Testing specific maladaptive beliefs, pre-treatment scores on cognitive confidence were associated with subjective cognitive impairments both pre and post-treatment, controlling for symptoms. Post-treatment metacognitive beliefs and post-treatment cognitive confidence were associated with post-treatment subjective cognitive impairments, controlling for pre-treatment impairments and pre-treatment metacognitive beliefs, as well as pre and post-scores on symptom measures.

**Conclusion:** This study reports associations between maladaptive metacognitive beliefs and subjective cognitive impairments in patients with chronic fatigue. Targeting metacognitive beliefs could prove an effective therapeutic intervention for subjective cognitive impairments in these patients.

## Introduction

Chronic fatigue syndrome (CFS) is a prevalent condition characterized by persistent mental and somatic fatigue (Loge et al., 1998; McCrone et al., 2003). Unfortunately, little ground has been made when it comes to understanding its etiology, agreeing on a case definition, or providing patients with efficacious treatments (Castell et al., 2011). This has led to symptom measures often being argued as the best way to classify and investigate these patients (Brurberg et al., 2014). One of several criteria for fulfilling a CFS case definition is reporting problems with forgetfulness, increased distractibility, and reduced mental alertness (Fukuda et al., 1994; Jason et al., 2004). These subjective cognitive complaints or impairments are part of all popular CFS classifications (Holmes et al., 1988; Sharpe et al., 1991) making them a hallmark of the disorder (Jason et al., 2004). Their importance is underlined by 90% of CFS patients rating problems with memory and concentration as a primary concern (Jason et al., 1999).

Memory and concentration are cognitive capabilities usually defined within the overarching term “executive control functioning” (Miyake and Friedman, 2012). Executive control includes many aspects such as the control of attention, the capacity to access and manipulate information in long-term memory, as well as monitoring current internal and external states (Funahashi, 2001). CFS patients report a perceived inability to perform cognitive tasks and some data indicates that CFS patients have a lowered performance on objective neuropsychological tests (Michiels and Cluydts, 2001). However, this objective bias appears to only be present when the tasks are highly demanding (Dobbs et al., 2001; Cook et al., 2007). As an example, performance differences between CFS patients and controls are reported on the modified Paced Auditory Serial Attention Test, but not for a simple number recognition task (Cook et al., 2007). Subjective cognitive impairments on the other hand, are consistently reported (Cockshell and Mathias, 2014).

Performing demanding cognitive tasks causes mental fatigue (Cook et al., 2007), which here refers to temporary depletion of cognitive resources. Within CFS, mental fatigue has documented effects on attention, working memory, and other executive control processes (Boksem and Tops, 2008). Hence, evaluation of predicted rewards and costs quickly becomes relevant. Most of us tend to loose motivation to perform when the energy costs are believed to outweigh rewards (Boksem and Tops, 2008). This means that a subjective cost-benefit analysis of mental fatigue, or subjective impairment, could affect patients' objective ability to efficiently attend to, store and retrieve information when solving mental tasks.

However, such linking of subjective and objective cognitive functioning in CFS is controversial and not straightforward (Cockshell and Mathias, 2014). The foremost example of this is lack of a consistent correlation between tests (Wearden and Appleby, 1997), and that both objective performance and perceived problems could be a result of, or influenced by, co-morbid symptoms such as pain, insomnia, depression, and anxiety (Cockshell and Mathias, 2014). A recent review argues that subjective and objective measures of cognitive complaints could in fact represent entirely different constructs in these patients (Cockshell and Mathias, 2014).

Subjective cognitive evaluation can be argued as a form of metacognitive monitoring (Shimamura, 2000). The concept of metacognitions has become increasingly popular in psychotherapy research, also within CFS (Fernie et al., 2015). Metacognitions are popularly defined as “beliefs about thinking” or “cognitions about cognition” (Wells, 2011), and metacognitive theory is concerned with excessive, sustained verbal thinking, and dwelling on negative emotions or symptoms in the form of rumination and/or worry (Nolen-Hoeksema, 2000; Wells, 2011). It is a novel perspective associated with symptom severity (Fernie et al., 2015), but targets “perception of” and “dwelling on” symptoms rather than objective changes to metabolism or nociception. Hence, intervening on metacognitions might offer a new treatment approach, especially when it comes to subjective cognitive evaluation.

This notion is substantiated by rumination and worry being cognitive processes associated with increased mental fatigue (Querstret and Cropley, 2012). Moreover, excessive worry and rumination creates and maintains a specific attention bias toward threats (Nolen-Hoeksema, 2000; Maren, 2007; Browning et al., 2010). This bias has within metacognitive theory been coined the “cognitive attentional syndrome” (CAS; Wells, 2011). It is there described as a metacognitive process, which maintains and exacerbates depression and anxiety. However, the CAS may prove relevant in CFS as well (Maher-Edwards et al., 2011; Fernie et al., 2015), especially considering data showing CFS patients interpreting more bodily sensations as sign of physical disease, compared to depressed and healthy controls (Dendy et al., 2001). Moreover, a study investigating such selective attention show CFS patients having a significant bias toward health-threatening information (Hou et al., 2008).

The theoretical model outlining the development and maintenance of CAS is the Self-Regulatory Executive Function (S-REF) model (Wells and Matthews, 1996; Wells, 2011). This model provides a conceptualization of how metacognitive and cognitive factors could be involved in the top-down control of disorders. A central premise in this model is that all individuals have positive and negative beliefs about thinking that influences their appraisals (e.g., “I must worry in order to be prepared” or “I cannot control my thoughts”). Another premise is that we have implicit procedural metacognitions or “thinking skills” such as heuristics that influence and shape both our cognition and behavior (Wells and Matthews, 1996; Wells, 2011).

Investigating subjective cognitive impairments in chronic fatigue and whether these are associated with dysfunctional metacognitive beliefs, may further our understanding and treatment of these complaints. As of now, there are no studies investigating this association, but one study showcased the potential relevance of maladaptive metacognitive beliefs in patients with CFS (Fernie et al., 2015).

It is particularly interesting to see if maladaptive metacognitive beliefs are associated with subjective cognitive complaints independent of symptoms. Subjective cognitive impairments have previously been reported to predict quality of life and function in CFS, irrespective of co-morbid symptoms (Ray et al., 1997).

Testing the independent effect of metacognitions is also important for treatment development. Several promising treatment manuals for metacognitive therapy exist, which could easily be adapted to fit CFS, either as a stand-alone therapy, or as an addition to graded exercise therapy or traditional cognitive therapy. Thus, an investigation of metacognitive beliefs and their association to subjective cognitive complaints in patients with chronic fatigue is warranted.

The main aim of this study is to investigate the association of metacognitive beliefs to subjective cognitive impairments in a population reporting chronic fatigue. We hypothesize that maladaptive metacognitive beliefs are associated with subjective cognitive impairments at baseline, controlling for symptoms of fatigue, pain, insomnia, depression, and anxiety. Moreover, we hypothesize that maladaptive metacognitive beliefs at the start of treatment will predict subjective cognitive impairments at treatment end. We also hypothesize that a reduction in maladaptive metacognitive beliefs during treatment is associated with less subjective cognitive impairments at treatment termination, controlling for the aforementioned symptoms.

## Methods

### Setting and participants

This study had a pre-post design and was conducted between January 2012 and June 2013 through a 3, 5-week in-patient, Return-To-Work (RTW) occupational rehabilitation program at Hysnes Rehabilitation Center, St. Olav's University Hospital, Trondheim, Norway. In Norway a work week includes working Monday through Friday, or 5 days of work of 7, 5 h each. A work week is then 37, 5 h in total. Since the program lasted 3 work weeks and 2 work days, it consisted of a total of 17, 7, 5-h workdays during which there was an active intervention from the institution. The intervention was shaped this way to emulate a normal work week making the transition to work life easier for the patients. The choice of an inpatient setting for this program was due to geographical challenges specific to Norway. However, the treatment delivered did not exceed that of a day-based outpatient program with regard to hours and available personnel (total availability and treatment was 6.5 h from 8:30 a.m. to 3:00 p.m.). For details about the intervention see Fimland et al. (2014).

Prior to the enrolment in this program, the patients had been referred from their general practitioner and examined by an outpatient multidisciplinary team consisting of a physician, a psychologist, and a physiotherapist. This team evaluated whether the referred patients met the requirements for participating in the RTW-program, which included, but were not limited to the inclusion and exclusion criteria for the current study. Before beginning the rehabilitation program, all patients were asked to complete different questionnaires through an internet-administrated self-report survey. At the end of the study, which coincided with the end of the rehabilitation program, the patients again answered several questionnaires.

The inclusion criteria for the study were age (18 to 59 years) and having been on sick leave for at least 8 weeks due to fatigue. Further, participants should have self-defined goals of increasing labor participation, to be adequately assessed and treated beforehand for any specific illnesses, and be able to attend a rehabilitation program from 8:30 to 3:00 p.m. all weekdays. The exclusion criteria were severe mental illness (ongoing mania, psychosis or suicidal ideation), substance abuse and addiction or pregnancy. Moreover, patients who could not communicate in Norwegian or who needed 24-h personal assistance were not eligible.

To be eligible for this study, participants had to report a score of five or more on the fatigue questionnaire, a cut-off validated through previous studies of Norwegian adults (Loge et al., 1998). Moreover, to be included in all the planned steps of analyses, the patients could not have any missing data on any of the independent variables or covariates targeted in the subsequent multivariable analyses. Hence, the study population consisted of patients meeting these criteria who upon inclusion in the program gave their informed consent.

The Regional Committee for Ethics in Medical Research approved this study and it was conducted in accordance with the declaration of Helsinki. The study was registered in clinicaltrials.gov (ID: NCT01568970).

### The rehabilitation program

Details on the rehabilitation program are published elsewhere (Fimland et al., 2014). In brief, the program used a combination of group treatment (8 patients per group) and individual approaches to facilitate rehabilitation. The participants were organized with activities through 7-h workdays and the program lasted for a total of 17 workdays with Acceptance and Commitment Therapy (ACT) as the overarching treatment model. Therapists targeted three areas of rehabilitation: mental training, physical training, and work-related problem solving.

### Measures

The participant's demographics were reported through a standardized set of questions validated for a large Norwegian cohort (Holmen et al., 2003), which is also detailed in previous studies (Jacobsen et al., 2014; Kallestad et al., 2015).

### Dependent variable

Subjective cognitive complaints were reported using The Everyday Memory Questionnaire-Revised (EMQ) (Royle and Lincoln, 2008). The EMQ-Revised is a 13-item measure with two main factors of retrieval and attentional tracking, reduced from an original 34-item questionnaire (Royle and Lincoln, 2008). Reliability tests on the EMQ have shown a strong internal reliability, with a Cronbachs' Alfa score of.89. Each question is rated on a 5-point scale ranging from A, scored as one—“Once or less in the last month,” to E, scored as five—“Once or more in a day.” These items are summed and give an ordinal scale with a range of 0 to 64.

### Independent variables and covariates

The Chalder Fatigue Questionnaire (Chalder et al., 1993) consists of eleven questions asking about physical and mental fatigue and is frequently used to measure symptoms in chronic fatigue patients. Each item has four response categories (0-4), which are scored bi-modally 0-0-1-1. When scored, the 11 items are summed and gives each participant a score on a scale of 0–11. This eleven-item scale has been validated for a Norwegian adult population with a cut-off on symptom intensity ≥5. Cronbach's alpha has been calculated for all items (range 0.88–0.90). Split half reliability has also been calculated (0.86 and 0.85, respectively) (Loge et al., 1998).

The Metacognitions Questionnaire-30 (MCQ-30; Wells and Cartwright-Hatton, 2004) asks about metacognitive beliefs through 30 items, compromising five subscales: (1) Positive beliefs about worry (e.g., “Worrying helps me cope”); (2) Negative beliefs about uncontrollability and danger of worry (e.g., “When I start worrying I cannot stop”); (3) Cognitive confidence (e.g., “I have a poor memory”); (4) Need to control thoughts (e.g., “Not being able to control my thoughts is a sign of weakness”); (5) Cognitive self-consciousness (e.g., “I pay close attention to the way my mind works”). Items are scored from 1 to 4 (“do not agree,” “agree slightly,” “agree moderately,” “agree very much”), and adding up individual subscale items scores the subscales. Cronbach's alpha coefficients for these subscales range from 0.72 to 0.93, with test-retest correlations of: 0.75 (total score), 0.79 (positive beliefs), 0.59 (uncontrollability/danger), 0.69 (cognitive confidence), 0.74 (need for control), and 0.87 (cognitive self-consciousness; Wells and Cartwright-Hatton, 2004).

The Hospital Anxiety and Depression Scale (HADS) (Zigmond and Snaith, 1983) asks about symptoms of *anxiety and depression*. The fourteen-item scale with each item ranging from 0 to 3 yields separate scores for anxiety and depression, which are then summed and used as an ordinal scale. In a review of HADS in Norwegian adults the correlations between the two subscales varied from 0.40 to 0.74 (mean 0.56). Cronbach's alpha for HADS-A varied from 0.68 to 0.93 (mean 0.83) and for HADS-D from 0.67 to 0.90 (mean 0.82; Bjelland et al., 2002).

The Insomnia Severity Index (Bastien et al., 2001; ISI) is a seven-item questionnaire assessing *sleep problems*. A 5-point (0–4) scale, rated difficulties falling asleep, night-time awakenings, early morning awakenings, impairment of daytime functioning due to sleep problems, notice ability of impairments, distress or worry caused by sleep difficulties, and dissatisfaction with sleep. The items were summed; giving a scale of 0–28, where ≥15 was used as a moderate cut-off indicating sleep problems. This cut-off has been validated in previous studies and the internal consistency of the ISI was reported to be 0.74 (Morin et al., 2011).

*Chronic pain* was measured with an item from Short Form-8 (Ware et al., 2001) asking “*How much bodily pain have you had the last week*?” (None, very mild, mild, moderate, severe, and very severe). This scale has been validated as a self-report measure of chronic pain in Norwegian populations. As this is a one-item measurement, alpha values are not applicable. The item has been shown to have an intra-class correlation coefficient of 0.66 (95% CI 0.65 to 0.67; Landmark et al., 2012).

### Statistical analysis

Demographical data from participants was analyzed using frequencies (dichotomous variables) and means (ordinal variables) with standard deviations. Paired *t*-tests were performed to investigate significant change from pre to post-treatment on subjective cognitive impairments, maladaptive metacognitions, insomnia, fatigue, pain, depression, and anxiety.

To test for associations between maladaptive metacognitive beliefs (MCQ-30) and subjective cognitive impairments at baseline, we performed two hierarchical linear regression models: First, using subjective cognitive impairments pre-treatment as a dependent variable we performed a hierarchical regression model with two steps. In step one, we entered participants' sex and age, and in step two we entered pre-treatment MCQ-30 sum total score. Then, we performed a hierarchical linear regression with three steps. In step one, we entered participants' sex and age, and in step two we entered pre-treatment scores on the five symptom measures. In step three we entered pre-treatment MCQ-30 sum total score.

Next, we performed a third hierarchical regression model testing the associations of the five subscales of MCQ-30 (cognitive confidence, need to control thoughts, positive beliefs, danger/uncontrollability and cognitive consciousness) with subjective cognitive impairments. In step one, we entered participants' sex and age, and in step two we entered pre-treatment scores on symptom measures. In step three we entered pre-treatment scores on cognitive confidence need to control thoughts, positive beliefs, danger/uncontrollability, and cognitive consciousness.

To test whether maladaptive metacognitive beliefs (MCQ-30) at treatment start were associated with subjective cognitive impairments at treatment termination, we performed a hierarchical linear regression using subjective cognitive impairments post-treatment as a dependent variable. In this regression model we entered sex and age in step one, in step two we entered pre-treatment values for subjective cognitive impairments. In step three we entered pre-treatment sum total scores for MCQ-30.

To test whether a reduction in maladaptive metacognitive beliefs (MCQ-30) were associated with change in subjective cognitive impairments, we added fourth step in the hierarchical linear regression using subjective cognitive impairments at treatment termination as the dependent variable. We entered sex and age in step one, in step two we entered pre-treatment scores for subjective cognitive impairments, in step three we entered pre-treatment sum total score for MCQ-30. In step four, we entered the MCQ-30 sum total score at treatment end.

To test whether maladaptive metacognitive beliefs (MCQ-30) was associated with subjective cognitive impairments (EMQ) controlling for levels of fatigue, pain, insomnia, depression, and anxiety, we performed a hierarchical linear regression using subjective cognitive impairments at treatment termination as the dependent variable. We entered sex and age in step one, in step two we entered pre-scores for EMQ, and in step three we entered pre-treatment sum total scores on MCQ-30. In step four we entered pre-treatment scores on the five symptom measures. In step five we entered post-treatment sum total scores for MCQ-30 and in step six we entered post-treatment scores on the five symptom measures.

To test whether the subscales of the maladaptive metacognitive beliefs (MCQ-30) questionnaire were associated with subjective cognitive impairments (EMQ) controlling for levels of fatigue, pain, insomnia, depression, and anxiety, we performed a hierarchical linear regression using subjective cognitive impairments at treatment termination as the dependent variable. We entered sex and age in step one, in step two we entered pre-scores for EMQ, and in step three we entered pre-treatment scores on the subscales of MCQ-30. In step four we entered pre-treatment scores on the five symptom measures. In step five we entered post-treatment scores on the subscales of MCQ-30 and in step six we entered post-treatment scores on the five symptom measures.

All analyses were performed using the Statistical Package for the Social Sciences (SPSS version 22.0; IBM Corporation, Armonk, NY, USA). In all regressions we estimated correlations and colinearity in order to ensure the validity of the models.

## Results

### Population demographics

One hundred and eighty-one participants were included in the current study. Of these 181 participants, 137 had no missing data, were available for multivariable analysis and *t*-tests, and represented the study population. All participants reported fatigue above the chosen cut off. They were predominantly female (80.3%) with a mean age of 43.6 (SD 9.4) years, and with education at a high school level or above (87.6%). For an overview of participant characteristics, see Table 1. Paired *t*-tests of change for subjective cognitive impairments and maladaptive metacognitive beliefs are presented in Table 2.

**Table 1:** **Characteristics of all included participants (*n* = 137) self-reporting clinically significant physical and mental fatigue when starting inpatient vocational rehabilitation**.

**Demographics**	**Male**	**Female**
Sex (n)	19.7% (27)	80.3% (110)
Age (SD)	41.0 (11.8)	41.1 (9.2)
Height—cm (SD)	180.4 (8.1)	166.8 (5.9)
Weight—kg (SD)	88.3 (15.7)	76.0 (16.4)
BMI (SD)	27.0 (4.0)	27.3 (5.6)
Diagnosed chronic fatigue in ICPC-2: A04 or P29 (n)	55.6% (15)	49.0% (54)
**RELATIONSHIP STATUS (n):**		
Married/Living with Partner (n)	66.6% (18)	70.6% (79)
Single/Divorced/Widowed (n)	44.4% (9)	29.4% (33)
**EDUCATION (n):**		
Less than High School	18.5% (5)	18.0% (20)
High School	40.7% (11)	35.1% (39)
Some College/University	29.6% (8)	40.5% (45)
Any College/University Degree	11.1% (3)	6.3% (7)
**PSYCHIATRIC DIAGNOSES (n):**		
SCID diagnosed Anxiety disorders (F40.1 F40.2 F41.0 F41.1 F41.2 F41.9 F45.2)	7.4% (2)	9.7% (11)
SCID diagnosed Depressive disorders (F32.0 F32.1 F.33.1 F34.1 F43.2)	7.4% (2)	7.1% (8)
**Self-reported symptoms above cut-off**	**Male**	**Female**
HADS Depression (n)	51.9% (14)	42.9% (48)
HADS Anxiety (n)	55.6% (15)	54.9% (62)
Chronic Pain (n)	59.3% (16)	78.8% (89)
Sleep Problems (n)	33.3% (9)	26.5% (30)

*Categorical characteristics are reported as percent (frequency) and ordinal characteristics as mean (standard deviation). Numbers may not add up to 137 because of missing data on some characteristics. Percentage is calculated as percentage of total n of the respective sex (male/female). F43.2 is categorized as a depressive disorder in this table, however, it could also be classified as an anxiety disorder as it has somewhat overlapping symptoms. Some participants have two SCID diagnoses and were categorized by the primary diagnosis*.

**Table 2:** **Averaged change on outcomes, metacognitive beliefs, fatigue, mental distress (including insomnia) and pain reported by participants' pre-post intervention**.

**Variable**	**Pre-treatment**		**Post-treatment**		**Paired samples** ***t*****-test**		
	**Mean**	***SD***	**Mean**	***SD***	***t***	***p***	***g***
**COGNITIVE MEASURES:**							
Subjective cognitive complaints (EMQ)	30.8	12.3	29.8	12.6	1.4	0.17	0.03
MCQ-30 total	53.8	12.2	51.0	11.2	3.9	< 0.001	0.2
**MCQ SUBSCALES:**							
Cognitive confidence	13.1	4.6	12.1	4.4	3.2	0.002	0.15
Positive beliefs	7.6	2.2	7.3	1.7	1.7	0.1	0.05
Cognitive consci.	11.8	3.4	11.8	3.5	−0.3	0.98	0.2
Uncontrollability	11.0	3.7	11.5	3.8	−2.0	0.05	0.2
Need to control thoughts	9.7	3.1	9.9	3.2	−0.9	0.35	0.2
**MENTAL DISTRESS, FATIGUE, AND PAIN:**							
Insomnia (ISI)	12.2	6.1	9.8	6.4	5.53	< 0.001	0.38
Pain (SF-8)	3.95	1.14	3.5	1.15	4.97	< 0.001	0.39
Depression (HADS)	7.1	3.8	4.9	3.4	8.0	< 0.001	0.65
Anxiety (HADS)	8.2	4.2	6.2	3.8	6.6	< 0.001	0.50
Fatigue (CFQ)	9.0	1.8	5.7	3.9	10.6	< 0.001	1.08

*All variables were significance tested with a paired t-test and degree of change was described as a Hedges g effect size*.

### Hypothesis 1: Maladaptive metacognitive beliefs are associated with subjective cognitive impairments at baseline

The sum score on maladaptive metacognitive beliefs (MCQ-30) at baseline was significantly associated with the subjective cognitive impairments (EMQ) sum score at baseline (*p* < *0.0001; t* = *4.5; B* = *0.34; 95% Confidence Interval of B (CI)* = *0.19, 0.49*), controlling for sex, age and pre-scores on symptom scales. Fatigue score pre-treatment (*p* < *0.0001; t* = *6.4; B* = *3; CI* = *2.1, 3.9*) and depression score pre-treatment (*p* = *0.03; t* = −*2.2; B* = −*0.53; CI* = −*1*, −*0.61*) were also significantly associated with EMQ sum score at baseline.

Investigating the subscales of MCQ-30, the subscale of cognitive confidence (*p* < *0.0001; t* = *8.2; B* = *1.4; CI* = *1.1, 1.8*) was significantly associated with the EMQ sum score, controlling for pre-treatment symptom scores, as was the pre-score on the chosen fatigue scale (*p* < *0.0001; t* = *3.6; B* = *1.7; CI* = *0.77, 2.6*). The subscales of positive beliefs, need to control thoughts, danger and cognitive consciousness were not. Zero order correlations between the self-report measures ranged from (−0.2 to 0.6), with cognitive confidence being the highest and need for thought control the lowest. Both collinearity tolerance (1.0–0.5) and variance inflation factor values (1.0–2.4) were acceptable in the regression model.

### Hypothesis 2: Maladaptive metacognitive beliefs pre-treatment are associated with subjective cognitive impairments at treatment termination

A hierarchical regression analysis showed that the pre-treatment sum total score on MCQ-30 was not associated with EMQ sum score post-treatment, when controlling for pre-scores on the EMQ (*p* = *0.77; t* = *0.29; B* = *0.17; CI* = −*0.1, 0.13*).

As pre-treatment cognitive confidence was the only subscale associated with pre-treatment score on EMQ, we performed an additional analysis looking specifically at this subscale. Pre-treatment scores on cognitive confidence were significantly associated with post-treatment scores on EMQ, controlling for scores on all symptom measures both pre and post-treatment (*p* < *0.0001; t* = −*3.8; B* = −*0.7; CI* = −*1.1*, −*0.35*).

### Hypothesis 3: A reduction in metacognitive beliefs post-treatment is associated with less subjective cognitive complaints at treatment termination

Results from a hierarchical regression showed that a reduction on MCQ-30 was significantly associated with a reduced post-treatment score on EMQ, controlling for pre-treatment MCQ-30 scores, fatigue scores (pre-post), pain (pre-post), insomnia severity (pre-post), anxiety and depression (pre-post) (*p* < *0.0001; t* = *3.6; B* = *0.35; CI* = *0.16, 0.5*). Analytic details of the regression are presented in Table 3.

**Table 3:** **Hierarchical regressions using everyday memory questionnaire (EMQ) post-treatment as a dependent variable and metacognitive questionnaire (MCQ) as an independent variable, controlling for pre-treatment values of EMQ, MCQ, fatigue, pain, insomnia, depressive symptoms and post-treatment values of fatigue, pain and depressive symptoms**.

**Variables**	**Beta Coefficient**	***P***	**95% Confidence Interval**	
			**Low**	**High**
**STEP 1:**				
Sex	−0.127	0.165	−9.421	1.628
Age	0.097	0.286	−0.119	0.400
**STEP 2:**				
Sex	−0.080	0.142	−5.753	0.838
Age	0.048	0.374	−0.085	0.224
Pre-treatment EMQ	0.798	0.000	0.691	0.906
**STEP 3:**				
Sex	−0.080	0.145	−5.761	0.857
Age	0.048	0.375	−0.086	0.225
Pre-treatment EMQ	0.793	0.000	0.678	0.908
Pre-treatment MCQ-30	0.016	0.785	−0.099	0.131
**STEP 4:**				
Sex	−2.761	0.125	−6.305	0.783
Age	0.086	0.288	−0.074	0.246
Pre-treatment EMQ	0.775	0.000	0.642	0.907
Pre-treatment MCQ-30	0.050	0.452	−0.081	0.182
Pre-treatment Fatigue	0.068	0.884	−0.850	0.986
Pre-treatment Anxiety	−0.204	0.315	−0.603	0.196
Pre-treatment Depression	−0.183	0.414	−0.626	0.260
Pre-treatment Pain	−0.237	0.707	−1.487	1.012
Pre-treatment Insomnia	0.169	0.189	−0.085	0.424
**STEP 5:**				
Sex	−2.337	0.158	−5.597	0.923
Age	0.095	0.204	−0.052	0.242
Pre-treatment EMQ	0.723	0.000	0.600	0.847
Pre-treatment MCQ-30	−0.207	0.014	−0.371	−0.044
Pre-treatment Fatigue	0.145	0.734	−0.699	0.989
Pre-treatment Anxiety	−0.185	0.320	−0.552	0.182
Pre-treatment Depression	−0.405	0.057	−0.823	0.013
Pre-treatment Pain	0.224	0.704	−0.941	1.389
Pre-treatment Insomnia	0.152	0.200	−0.082	0.385
Post-treatment MCQ-30	0.411	0.000	0.235	0.587
**STEP 6:**				
Sex	−2.330	0.162	−5.615	0.954
Age	0.054	0.459	−0.091	0.200
Pre-treatment EMQ	0.728	0.000	0.608	0.849
Pre-treatment MCQ-30	−0.138	0.098	−0.302	0.026
Pre-treatment Fatigue	−0.081	0.859	−0.983	0.821
Pre-treatment Anxiety	0.048	0.822	−0.375	0.471
Pre-treatment Depression	−0.515	0.030	−0.981	−0.050
Pre-treatment Pain	−0.342	0.613	−1.678	0.993
Pre-treatment Insomnia	−0.041	0.783	−0.332	0.251
Post-treatment MCQ-30	0.347	0.000	0.156	0.538
Post-treatment Fatigue	0.197	0.334	−0.205	0.599
Post-treatment Pain	1.350	0.054	−0.026	2.727
Post-treatment Insomnia	0.182	0.239	−0.123	0.487
Post-treatment Anxiety	−0.581	0.048	−1.156	−0.006
Post-treatment Depression	0.517	0.095	−0.092	1.125

*Everyday memory questionnaire—Revised (EMQ); Metacognitive beliefs questionnaire (MCQ)*.

As pre-treatment cognitive confidence was associated with pre-treatment and post-treatment score on EMQ, we again performed an additional analysis looking specifically at this subscale. Post-treatment scores on cognitive confidence were significantly associated with post-treatment scores on EMQ, controlling for scores on all symptom measures both pre and post-treatment (*p* < *0.0001; t* = *6.0; B* = *1.2; CI* = *0.8, 1.7*).

## Discussion

This is the first study to report associations between maladaptive metacognitive beliefs and subjective cognitive impairments in chronically fatigued patients. Maladaptive metacognitive beliefs were associated with subjective cognitive complaints at baseline. Investigating the subscales of MCQ-30, only the scale asking beliefs about cognitive confidence had pre-treatment scores associated with pre-treatment subjective cognitive impairments. When looking at whether maladaptive metacognitive beliefs at baseline predicted subjective impairments at treatment end, we found that the sum of MCQ-30 did not predict subjective ratings. However, baseline scores on the subscale of cognitive confidence did predict subjective impairments at treatment end. Moreover, when testing if a reduction in sum total maladaptive metacognitive beliefs predicted less subjective cognitive impairments at treatment termination, we found a significant association that remained when controlling for baseline subjective cognitive complaints, and pre-post scores on fatigue, pain, insomnia, depression and anxiety. This was also the case for a reduction in post-treatment values on the cognitive confidence subscale, indicating that this subscale is particularly relevant in subjective cognitive impairments.

Our finding that baseline metacognitive beliefs concerning cognitive confidence were associated with baseline scores on subjective cognitive complaints is new. According to metacognitive theory, metacognitive beliefs about the lack of cognitive confidence will stop adaptive cognitive coping strategies when patients are feeling mental fatigue. Indeed, maladaptive metacognitive beliefs about cognitive confidence have been previously been associated with symptom severity in CFS patients (Fernie et al., 2015). It is not unlikely then that this association with symptom severity is related to patients' subjective cognitive impairments. This claim is substantiated by memory evaluations, such as our judgments of learning abilities being construed as a specific form of metacognitive monitoring (Shimamura, 2000).

The current results may have interesting clinical implications. According to recent theorizing in metacognition, cognitive processes are split into two interrelated levels: the meta-level and the object level (Wells, 2011). The meta-level contains a cognitive model of the object level, organized according to certain metacognitive principles. The meta-level is continuously updated by bottom-up information, and in return controls the object level by providing top-down input, initiating and terminating actions performed by the object level, here understood as an experience of memory or concentration failure. Thus, metacognitive regulation is a meta-level system that modulates cognitive processes at the lower level. It adds flexibility to cognitive processes, making them less dependent on external cues (Wells, 2011). Metacognitions also include self-reflection. The ability to recognize ones own maladaptive behavior and perpetuation of symptoms through rumination and worry is an important metacognitive skill (Wells, 2011). Self-reflection is largely a product of early life experiences (Dimaggio et al., 2010), and it is difficult to exclude effects from these experiences in the current study. However, given that one does not have an adequate metacognitive skill to guide actions when experiencing fatigue, this could be a marker for increased risk for developing long-term fatigue. Thus, targeting cognitive confidence both as a screening in patients initially reporting fatigue, subsequently targeting it through metacognitive therapy, could provide us with both a screening tool and a therapeutic intervention.

Our finding that cognitive confidence at baseline predicted subjective cognitive complaints at treatment end is also interesting in this regard. Previous data have shown that CFS patient's *objective* cognitive abilities will vary over a span of 10 weeks (Fuentes et al., 2001). It is perhaps indicated here that subjective cognitive evaluations are more stable constructs and therefore a more apt target for intervention. The current results suggest that an intervention on maladaptive metacognitions about cognitive confidence would positively influence subjective evaluations over time. This would potentially alleviate a concern that 90% of CFS patients rate as primary and debilitating.

The main finding in this study was that a reduction in dysfunctional metacognitive beliefs during treatment was associated with less subjective cognitive impairments at treatment termination. This relationship was not affected by symptoms of fatigue, pain, insomnia, anxiety or depression. Previous studies have attributed subjective cognitive impairments in CFS to the level of depressive symptoms in patients (Wearden and Appleby, 1997; Cockshell and Mathias, 2014). While depressive symptoms and anxiety also showed a significant association with subjective cognitive complaints in the current study population, they did not influence the unique association with maladaptive metacognitive beliefs.

However, the reported relationship between depressive symptoms, anxiety, and subjective cognitive complaints does add a second argument for investigating metacognitions further. Metacognitive therapy has previously shown promising results with depressed patients lacking success from traditional cognitive behavioral therapy (CBT; Wells et al., 2012). Lack of success with CBT is also the case for many chronic fatigue patients (Castell et al., 2011), and metacognitive therapy could be a feasible option for such cases. Moreover, metacognitions have been argued as highly relevant in the development and maintenance of general anxiety disorder (Wells, 2005), a diagnosis often comorbid with CFS (Fischler et al., 1997).

Of particular relevance to the current results is a recent text comparing subjective vs. objective cognitive impairment in chronic fatigue syndrome (Cockshell and Mathias, 2014). The authors argue that these are different concepts entirely. It seems that subjective impairment is somewhat imprecise and more generic, while objective impairments are closely linked to task difficulty (Cockshell and Mathias, 2014).

Considering this, it is tempting to further speculate on how subjective cognitive complaints could be a prime target for intervention. Perseverative thinking in the form of subjective cognitive complaints may lead to physiological changes or prolonged activation, and that in turn might change the patients experience of fatigue and/or disrupt recovery (Brosschot et al., 2006). Current stress theory argues that by targeting such perseverative thinking, we might be able to ameliorate not only subjective cognitive complaints, but also the intensity and frequency of fatigue symptoms (Brosschot et al., 2006).

It is important to note that this study used an ACT intervention, not metacognitive therapy. While our results imply that targeting metacognitive beliefs through metacognitive therapy is perhaps not necessary to change them, it does not answer whether using a therapy specifically focused on changing metacognitive beliefs could increase this change. The reported change in this study is minor, and it could very well be that a targeted metacognitive therapy would have made more impact on the patients'.

## Limitations

The current study has some limitations, the foremost being the lack of a control group. Another limitation is that outcome variables are based on self-report and not clinical or objective evaluations. Self-reported fatigue on the Chalder Fatigue Questionnaire was used as a cut-off for fatigue, opposed to a clinical examination, semi-structured interviews or fitness tests. Thus, the participants' results may not be generalizable to the group of patients who meet the full diagnostic criteria for CFS. However, the mean pre-treatment scores on Chalder Fatigue Scale are in line with other studies of patients with diagnosed CFS (White et al., 2011), and systematic reviews have argued that symptom measures are the best way to evaluate interventions in chronic fatigue (Brurberg et al., 2014). We did not have data on menopause/perimenopause status in our particpants, which could be considered a limitation since this can affect variables measured. It is also a limitation that subjective cognitive impairments were assessed by a questionnaire not an semi-structured interview, however, this is a common way of estimating such impairments. Moreover, with approximately 10% of participants reporting an emotional disorder on SCID-I, this might influence the data on maladaptive metacognitions, as these are prevalent in anxiety and depression. Finally, it is a limitation that 27% of patients' were excluded because of missing data, and this may have influenced our results.

## Conclusion

This non-controlled study reports significant associations between dysfunctional metacognitive beliefs and subjective cognitive impairments in chronically fatigued patients. These associations highlight the potential importance of targeting maladaptive metacognitive beliefs in chronic fatigue. Finally, our results give cause to investigate metacognitive therapy as a potential supplemental or novel psychological treatment for CFS.

## Author contributions

We all have participated in the conception of this report and have assisted in revising the manuscript for important intellectual content. We have all provided final approval of the enclosed report. More specifically: JA assisted with conceptual framework and writing as well as organized the data collection. PB was the project leader and essential in data collection as well as writing the methods and introduction. NL co-developed the experimental design, study aims and contributed to the discussion. TS supervised the entire production of the manuscript, co-developed the design, helped write the study aims and contributed to the introduction, results and discussion.

## Funding

This study was done using government funding through the Central Norway Regional Health Authority. The funders had no role in study design, data collection and analysis, decision to publish, or preparation of the manuscript.

### Conflict of interest statement

The authors declare that the research was conducted in the absence of any commercial or financial relationships that could be construed as a potential conflict of interest.
